# Supplementary material for: Identification of Freezing-Responsive microRNAs and Their Targets in Chinese Jujube by Small RNA and Degradome Sequencing
Source: Int J Mol Sci. 2026 Sep 4;27(17):7912. doi: 10.3390/ijms27177912 (PMC13566890; doi:10.3390/ijms27177912)
Supplement: Supplementary file 1 [file ijms-27-07912-s001.zip › Supplementary Material S2/novel_miR_7.pdf]

Provisional ID : novel_miR_7
Score total : 0.6
Score for star read(s) : -1.3
Score for read counts : 0
Score for mfe : 0.3
Score for randfold : 1.6
Score for cons. seed :
Total read count : 95
Mature read count : 95
Loop read count : 0
Star read count : 0

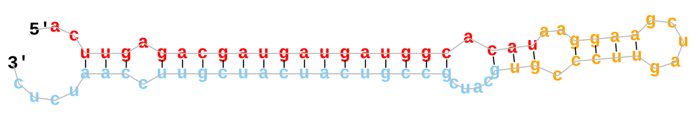

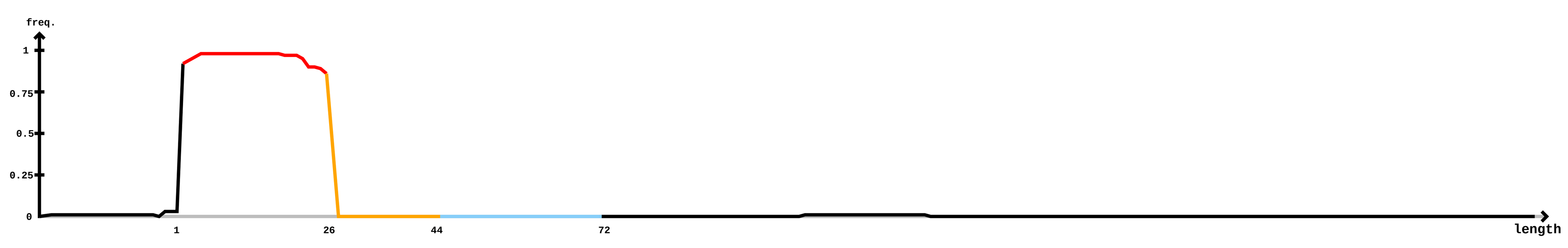

Mature Star

```
5'- augguacaagcucgaagccauuacuugagacgaugaugauggcacauaaggaagcuaguucccgugcaucgccgucaucaucguuccaaucucuauccaaauugguggaaaugaccauuaggaacauugaugaccuacaaucucuuccacaagaaggugugggccuaccucucaucucuccaaucucuucagauuagcaguugccccaauuugacucauugccagaaucuaucggcaaccucucaucaucu -3'  exp
    (((((..........)))))...(((.((((((((((((((.(((..((((.....)))).)))....))))))))))))))).)))........((((((((((((.((((....((((......(((........)))....(((((......)))))))))....))))...))))((((....))))..((....)).))))))))).....(((((.((.....)))))))............  reads  mm  sample
    augguacaagcucgaagc........................................................................................................................................................................................................................................  1  0  LCK-3
    ...................auuacuugGgacgaugauga...................................................................................................................................................................................................................  1  1  LT-1
    ...................auuacuugagacgaugaugaugg................................................................................................................................................................................................................  2  0  LT-2
    ......................acuugGgacgaugaugauggc...............................................................................................................................................................................................................  2  1  LT-1
    ......................acuugagacgaugaugauggc...............................................................................................................................................................................................................  1  0  LT-1
    ......................acuugagacgaugCugauggcacau...........................................................................................................................................................................................................  1  1  LCK-2
    ......................acuugagacgaugaugauggcacau...........................................................................................................................................................................................................  44  0  LCK-1
    ......................acuugagacgaugaugauggcacau...........................................................................................................................................................................................................  41  0  LCK-2
    .......................cuugGgacgaugaugauggcaca............................................................................................................................................................................................................  2  1  LT-3
    ........................uugGgacgaugaugauggc...............................................................................................................................................................................................................  2  1  JCK-3
    .........................ugagacgaugaugauggcac.............................................................................................................................................................................................................  1  0  LT-3
    .........................ugGgacgaugaugauggcaca............................................................................................................................................................................................................  1  1  JCK-2
    ..............................................................................................................................uugaugaccuacaaucucuuc.......................................................................................................  1  0  JCK-3
```
